# Developing an expanded vector control toolbox for malaria elimination

**DOI:** 10.1136/bmjgh-2016-000211

**Published:** 2017-04-26

**Authors:** Gerry F Killeen, Allison Tatarsky, Abdoulaye Diabate, Carlos J Chaccour, John M Marshall, Fredros O Okumu, Shannon Brunner, Gretchen Newby, Yasmin A Williams, David Malone, Lucy S Tusting, Roland D Gosling

**Affiliations:** 1 Environmental Health and Ecological Sciences Department, Ifakara Health Institute, United Republic of Tanzania; 2 Department of Vector Biology, Liverpool School of Tropical Medicine, Liverpool, UK; 3 Malaria Elimination Initiative, Global Health Group, University of California, San Francisco, California, USA; 4 Institut de Recherche en Sciences de la Santé/Centre Muraz, Bobo-Dioulasso, Burkina Faso; 5 Instituto de Salud Global, Barcelona Centre for International Health Research (CRESIB), Hospital Clínic, Universitat de Barcelona, Barcelona, Spain; 6 Instituto de Salud Tropical, Universidad de Navarra, Pamplona, Spain; 7 Divisions of Biostatistics and Epidemiology, School of Public Health, University of California, Berkeley, California, USA; 8 School of Public Health, University of the Witwatersrand, Johannesburg, South Africa; 9 Innovative Vector Control Consortium, Liverpool, UK; 10 Oxford Big Data Institute, Li Ka Shing Centre for Health Information and Discovery, University of Oxford, Oxford, UK

## Abstract

Vector control using long-lasting insecticidal nets (LLINs) and indoor residual spraying (IRS) accounts for most of the malaria burden reductions achieved recently in low and middle-income countries (LMICs). LLINs and IRS are highly effective, but are insufficient to eliminate malaria transmission in many settings because of operational constraints, growing resistance to available insecticides and mosquitoes that behaviourally avoid contact with these interventions. However, a number of substantive opportunities now exist for rapidly developing and implementing more diverse, effective and sustainable malaria vector control strategies for LMICs. For example, mosquito control in high-income countries is predominantly achieved with a combination of mosquito-proofed housing and environmental management, supplemented with large-scale insecticide applications to larval habitats and outdoor spaces that kill off vector populations en masse, but all these interventions remain underused in LMICs. Programmatic development and evaluation of decentralised, locally managed systems for delivering these proactive mosquito population abatement practices in LMICs could therefore enable broader scale-up. Furthermore, a diverse range of emerging or repurposed technologies are becoming available for targeting mosquitoes when they enter houses, feed outdoors, attack livestock, feed on sugar or aggregate into mating swarms. Global policy must now be realigned to mobilise the political and financial support necessary to exploit these opportunities over the decade ahead, so that national malaria control and elimination programmes can access a much broader, more effective set of vector control interventions.

**Key questions**

**What is already known about this topic?**

- Vector control in low and middle-income countries (LMICs), using long-lasting insecticidal nets (LLINs) and indoor residual spraying (IRS), accounts for most of the unprecedented malaria burden reductions achieved in the 21st century.
- LLINs and IRS are highly effective in LMICs, but are insufficient to eliminate malaria transmission in many settings because of operational constraints, mosquitoes that behaviourally avoid contact with them inside houses and growing resistance to available insecticides.
- However, mosquito control in high-income countries (HICs) is predominantly achieved with a combination of long-standing high coverage with mosquito-proofed housing and environmental management, supplemented with proactive, large-scale insecticide applications to larval habitats and outdoor spaces that kill off vector populations en masse.
- In contrast with the prescriptive, centralised global recommendation of LLINs and IRS as ubiquitous first-choice vector control tools for LMICs, the more aggressive, area-wide population suppression practices of HICs are idiosyncratically tailored to local conditions by decentralised mosquito abatement programmes, which are governed, funded and managed at the local level.

**What are the new findings?**

- A number of existing technologies are available that remain underdeveloped or underexploited, which could be rapidly mobilised to enable implementation of far more diverse, effective and sustainable malaria vector control strategies in LMICs.
- Where sufficient implementation capacity exists, and human population density is high enough to make the cost per person protected affordable, systems for vertical, proactive, locally managed delivery of mosquito population abatement technologies already used extensively in HICs should be developed and evaluated in LMICs. Experiences from programmes in HICs may be selectively leveraged wherever appropriate in LMIC contexts.
- Furthermore, a diverse range of repurposed and emerging technologies for targeting mosquitoes when they enter houses, feed outdoors, attack livestock, feed on sugar or aggregate into mating swarms are becoming available. These new technological options could all be developed into programmatically scalable vector control tools within the decade ahead and provide unprecedented opportunities for more effective suppression of malaria transmission in LMICs. Many of these technologies could be delivered horizontally, making them practically applicable even in settings with weak implementation capacity.

**Recommendations for policy**

- Global policy must now fully and consistently realign with both the programmatic needs and biological realities of malaria vector control, to prioritise accelerated development of these diverse options for malaria vector control in LMICs.
- Developing such an expanded toolbox for malaria vector control will require investment in product and system development, high-quality evaluations of efficacy and effectiveness, and operational research to define best practices for programmatic use of these additional interventions.

## Introduction

Vector control with long-lasting insecticidal nets (LLINs) and indoor residual spraying (IRS) accounts for an estimated 78% of the 663 million malaria cases averted globally since 2000.^1^ Despite these achievements, over 214 million malaria cases and 438 000 malaria-attributable deaths occurred in 2014.^2^ There are renewed calls for malaria eradication by 2040 and new bold global targets for malaria elimination: elimination from four southern African countries by 2020, a malaria-free Asia-Pacific by 2030,^3^ reductions in malaria incidence by 90% globally, and elimination in 35 countries by 2030.^4^ While IRS and LLINs provide the backbone of malaria control and elimination efforts in low-income and middle countries (LMICs) today, more aggressive approaches to vector control will be needed to achieve these ambitious future goals.^5^ ^6^ In countries that have successfully eliminated malaria, including high-income countries (HICs) such as the USA and Australia,^7^ ^8^ as well as lower income countries such as Mauritius, Sri Lanka and Turkey, mosquito populations were suppressed using more integrated vector control models, including multiple measures attacking different stages of the mosquito life cycle.^9–11^ To accelerate progress towards elimination, it is critical to revisit these existing methods, and to add new and emerging technologies that target different mosquito behaviours and life stages, so that low-income malaria-endemic countries can avail of a much larger arsenal of effective vector control options.

LLINs and IRS have been highly effective interventions in LMICs,^1^ but they have fundamental limitations, including (1) their vulnerability to selection for insecticide resistance,^12^ ^13^ (2) their reliance on population-wide human compliance for operational effectiveness,^14^ ^15^ (3) considerable cost,^2^ ^16–18^ and (4) important biological constraints to their efficacy caused by mosquitoes that feed on humans and/or animals outdoors, rest outdoors, or enter houses but then rapidly exit from them without being exposed to insecticides.^5^ ^6^ ^19^

Resistance to all four classes of insecticide available for public health, especially the pyrethroids we rely on for LLINs, is now prevalent across Africa.^12^ ^13^ New chemical insecticides are expected to enter the market over the next few years, but these may be similarly vulnerable to selection for physiological resistance if used as single active ingredients.^12^ ^13^ Improved deployment formats are needed to target insecticides more efficiently,^20–22^ so that mosaics, rotations or combinations,^23^ possibly including biological agents,^24^ can be affordably applied. For now, IRS is the only recommended alternative to LLINS for applying insecticides within houses, and most available alternatives to pyrethroids are far more expensive, resulting in slow uptake^16^ and contraction of IRS coverage wherever they have been adopted.^17^ As a result of all these financial and practical limitations, only 31% of African households have sufficient LLINs^25^ and global IRS coverage has shrunk to only 3.4% of the world's at-risk population.^2^

**Figure 1:**
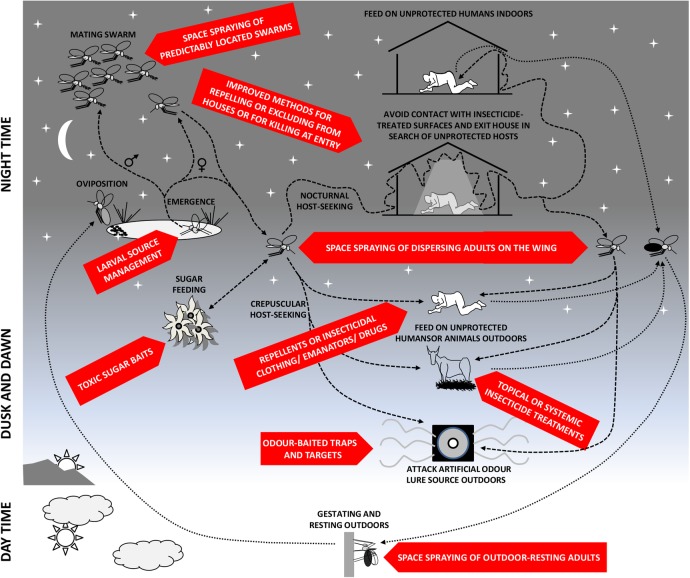
Schematic illustration of malaria vector mosquito life histories, highlighting the most important behaviours that mediate residual transmission of malaria despite high coverage with long-lasting insecticidal nets and indoor residual spraying,^5^ ^19^ as well as the many intervention opportunities that remain to be exploited with existing or emerging vector control methods. This figure has been updated relative to a previous version,^5^ to reflect evidence for the inclusion of additional intervention options, specifically odour-baited traps and targets for killing host-seeking mosquitoes (box 2), as well as targeted space spraying of mosquitoes (box 1), especially when they aggregate into mating swarms (box 3).

The impacts of LLINs and IRS are also biologically limited by their reliance on strong vector behavioural preferences for resting or biting in houses, usually associated with frequent feeding on humans.^5^ ^6^ ^19^ Wherever vectors exist that feed on animals, rest and/or feed outdoors, or can enter houses but rapidly exit again, malaria transmission is likely to persist despite a scale-up of LLINs and IRS, a phenomenon referred to as *residual* transmission (figure 1). In areas with self-sustaining levels of residual transmission, elimination of malaria cannot be achieved with LLINs and/or IRS alone, even if applied at universal coverage against a fully insecticide-susceptible vector population.^5^ ^6^ ^19^ ^26^ Reducing malaria transmission to levels where the rate of reinfection is low enough to eliminate parasite reservoirs from humans will require improved protection against human-biting mosquitoes, as well as more broadly effective population control of all major vector species, regardless of their diverse behavioural traits.^5^ ^6^ ^19^

In this analysis, we outline immediate opportunities for developing and implementing more aggressive malaria vector control strategies in LMICs, by leveraging transferable programmatic experiences from HICs with existing technologies, as well as exploiting repurposed and emerging new technologies. Additional new technologies include autodissemination of larvicides,^27^ genetic control,^28^ biological control^29^ and endectocides (systemic insecticides that are delivered to the tissues of target animals through oral, injectable or implant formulations) for humans,^30^ ^31^ but these are unlikely to be ready for programmatic assessment^19^ in <10 years. Here, we focus selectively on lower hanging fruit that could be feasibly deployed at scale by national malaria control programmes within the decade immediately ahead.

### Institutionalising robust delivery systems for existing mosquito population suppression technologies

Mosquito control in HICs has been predominantly achieved through a combination of mosquito-proofed housing and environmental management, supplemented with frequent, large-scale insecticide applications to larval habitats and outdoor spaces, to kill off vector populations en masse.^7^ ^8^ ^32^ ^33^ Some caution is required when considering the success of these HIC mosquito abatement programmes, because they have been conducted in less challenging temperate climates, where less efficient vectors bite humans too infrequently to mediate intense malaria transmission.^6^ Also, many of their routine operational practices will not be directly transferable to more financially constrained LMIC contexts, the needs of which are epidemiologically and ecologically diverse. Nevertheless, these interventions have been so successful that their malaria elimination function is often taken for granted, and many of the technologies or experiences may be relevant to malaria control and elimination in LMICs.

Many well-established and highly effective mosquito control technologies that have been applied in HICs for decades^7^ ^8^ remain to be widely adopted in LMICs because the necessary delivery systems and guidance have yet to be developed.^5^ Most HIC mosquito control programmes predominantly rely on large-scale larval source management (LSM) interventions to prevent the emergence of adult mosquitoes, complemented by space spraying of insecticides to tackle adult vector mosquitoes that do emerge (box 1, figure 1). Delivery of such proactive vector population control approaches typically requires vertical but decentralised delivery systems, managed locally by technical specialists, such as vector biologists, engineers and planners.^7^ ^8^ While HIC mosquito control programmes are predominantly staffed by such advanced specialists, such cadres are much sparser in LMICs, so the institutional structures and operational processes must be tailored accordingly.^9^ ^34^ Encouragingly, several examples of active, surveillance-based, vertically delivered local mosquito control programmes do exist in LMICs,^11^ operating at costs that are comparable with universal coverage of LLINs and IRS.^35–37^ Across settings, key features contributing to effective mosquito control systems include strong governance, dedicated financing, and decentralised management, robust entomological surveillance, and adaptive design of locally tailored intervention packages.

Box 1 **Existing vertically delivered mass population suppression technologies that have been underexploited in low-income and middle-income countries (LMICs)**

- *Larval source management (LSM) to prevent emergence of adult mosquitoes*: The mainstay of most mosquito control programmes in high-income countries (HICs) is aggressive control of immature mosquitoes in aquatic habitats through LSM, which includes all forms of environmental management, biological control and/or regular larvicide application that prevent immature aquatic stages of mosquitoes from emerging as adults.^9^ ^10^ The WHO currently recommends larviciding as a supplement to long-lasting insecticidal nets and indoor residual spraying in LMICs where larval habitats are *few, fixed and findable*, a situation for which supporting evidence of success in LMICs already exists.^9^ ^10^ However, given the advances in application technologies (eg, improved aerial and hand application systems) and emerging technologies for remotely identifying larval habitats, both driven by HIC markets over recent decades, LSM interventions may now become feasible in many LMIC contexts where it would previously have been considered unrealistic or unaffordable.
- *Space spraying to kill flying and resting adult mosquitoes*: Ground or aerial delivery of insecticides in the form of fine sprays, with small droplet sizes that remain suspended in the air for long periods, can kill mosquitoes which are resting or flying in the targeted time and place.^47^ This practice is often referred to as *space spraying* or *fogging*, and is a mainstay of HIC mosquito control programmes as a response to disease outbreaks and/or increase in mosquito abundance.^7^ ^8^ However, it has also been used in some LMIC settings, including countries like Turkey, Mauritius and Sri Lanka that have recently achieved malaria elimination.^11^ In its first elimination attempt, Haiti used aerial space spray to control *Anopheles* populations and reduce malaria transmission, and ground-based space spraying has been employed successfully for malaria vector control in India, Tanzania and El Salvador.^47–49^

The LSM and space-spraying interventions these programmes rely on are both area-wide interventions, so their application costs depend on the size of the catchment to be treated. Thus, the denser the human population, the more cost-efficient these measures become per person protected. With ongoing population growth, urbanisation and democratisation, development of such vertical but decentralised mosquito control programmes becomes an increasingly viable solution for many LMICs. Even now, ∼25% of the world's at-risk population already lives at densities of at least 1000 people per square kilometre, matching the sparsest population density at which LSM has already proven efficacious in Africa, albeit in the form of a district-scale research project rather than a larger programmatic evaluation.^38^

### Adapting and repurposing existing technologies to target vulnerable behaviours of adult mosquitoes

The remaining 75% of the world's malaria-prone population is probably too sparsely distributed to support specialist vertical programmes for active mosquito abatement. Therefore, other technological solutions requiring less advanced delivery capacity will also be needed.

Fortunately, a number of existing technologies are available for targeting adult mosquito blood-feeding behaviours, which could be readily adapted or repurposed for malaria vector control, and conveniently delivered through existing, non-specialist, horizontal distribution systems in LMICs (box 2, figure 1). While IRS and LLINs have been used to great effect^1^ ^2^ as affordable approaches to protecting sleeping and living spaces, they should ultimately be superseded by mosquito-proofed housing. However, considerable fractions of malaria transmission occur outdoors^5^ or within open housing designs that lack solid walls, much less a door. Insecticide-treated clothing or emanators for vapour-phase insecticides will therefore be needed to extend protection into the outdoor environment. Also, veterinary insecticides or mosquito traps baited with synthetic host odours may be used to achieve population suppression of outdoor-biting mosquito species. Indeed, such mass population abatement will most likely be essential to eliminate and prevent the reintroduction of malaria anywhere that vectors feed often enough on humans to mediate intense residual malaria transmission but also often enough on animals to evade population control with human-targeted interventions alone.^6^

Box 2 **Broadening horizontally delivered options for targeting blood-seeking adult mosquitoes**

- *Improving and extending physical protection of houses and peridomestic spaces*:^50^ Permanent housing modifications, such as window screening, sealed eaves and closed ceilings, can elicit remarkably high user acceptability, uptake and willingness to pay.^51^ Furthermore, mobile mosquito-proofed shelters may extend this approach to migrant lifestyles.^52^ In settings where long-lasting insecticidal nets (LLINs) and/or indoor residual spraying (IRS) previously performed mosquito population suppression functions,^6^ insecticide treatments for such screening materials are readily available.^53^ ^54^ Furthermore, some remarkably simple modifications to houses can turn them into lethal mosquito traps with^20–22^ or without^55^ insecticide. Critically, even these new formats that do require insecticide need far less active ingredient per household continuously protected,^20–22^ so implementation of the Global Plan for Insecticide Resistance Management^23^ may actually become affordable in practice.
- *Extending coverage with solid-phase contact insecticides by treating clothing*: Treating even the most basic garments and bed clothes with contact insecticides has long been known to protect against malaria exposure indoors and outdoors.^56^ ^57^ However, this approach can be limited by incomplete body coverage of many clothing practices in tropical climates, as well as restriction to a single pyrethroid insecticide (permethrin) which is safe enough for direct skin contact. While it might be possible to address the former limitation by supplementing with topical repellents applied to exposed skin,^58^ these only provide short-lived protection and require frequent reapplications, so they may be too expensive and impractical for effective, continuous, indefinite use in many low-income and middle-income country (LMIC) contexts.^59^ Furthermore, their active ingredients are often actually *irritants* rather than *repellents* in the strict sense,^60^ so they can exacerbate existing inequities by diverting mosquitoes to feed on unprotected non-users nearby^61^ ^62^ and undermine the impact of existing lethal interventions.^41^ ^63^
- *Vapour-phase insecticides for protecting open spaces*: Devices which emanate vapour from volatile insecticides or repellents into the air are already widely used in HICs for protection of open spaces where people are usually awake and active.^64^ ^65^ Encouragingly, one of the most widely used of these existing vapour-phase insecticides has recently been reformulated into low-technology emanator formats that provide protection lasting several months, at costs that should be affordable even in LMICs.^66–68^ While transfluthrin, and several similarly volatile pyrethroids, are often described as *spatial repellents*,^60^ ^69^ they can actually incapacitate^65^ or even kill mosquitoes.^70^ They are therefore described here as *vapour-phase insecticides*, to distinguish them from repellents and irritants in the strict sense, which may be associated with considerable limitations, disadvantages and risks.^41^ ^61–63^
- *Odour-baited traps targeting host-seeking mosquitoes*: Recent large-scale epidemiological trials have conclusively demonstrated mass suppression of the notoriously efficient African vector *Anopheles funestus*, and dramatically reduced malaria transmission by this widely distributed mosquito.^71^ These solar-powered traps are entirely self-sufficient in terms of electrical power and also provide sufficient surplus to provide household lighting and charge mobile phones. Given these direct benefits at the household level, irrespective of the impact on mosquitoes or malaria transmission, it may therefore be possible to distribute such traps programmatically through horizontal delivery mechanisms. Efficacy against a wider diversity of vector species will, however, require the development of affordable sources of carbon dioxide, or low-bulk substitutes for it.^72^
- *Veterinary insecticides to target vectors feeding on livestock*: Most malaria vector species prefer animals over humans^5^ ^6^ and are particularly dependent on cattle as a source of blood.^73^ A diverse range of advanced veterinary insecticide products exist for treating livestock animals, many of which could be readily repurposed for population suppression of malaria vectors through existing LMIC agricultural extension and market subsidy systems.^44^ ^74^ Targeting insecticide treatments to livestock can achieve the same kind of population suppression of mosquitoes which usually feed on animals,^75^ in the same way that LLINs and IRS do^15^ for human-specialised vectors.^6^ ^41^ ^76^ A particularly attractive aspect of targeting malaria vectors when they attack livestock is that there are already strong markets, subsidies, delivery systems and market intervention experience with these products in LMICS.^77^

Beyond the well-understood blood-feeding behaviours that are most obvious as targets for mosquito control, other behaviours that are critical to mosquito survival can be targets for malaria vector control (figure 1 and box 3). While female mosquitoes need blood from animals to develop their eggs to maturity, they also feed on plant sugar sources to maintain their energetic requirements. Male mosquitoes also feed on sugar, so both sexes can be targeted with attractive toxic sugar baits to deplete local vector populations. Aggregation of male mosquitoes into swarms, to attract and compete for female mosquitoes, presents another critical mosquito behaviour that may be targeted with ground-based insecticide sprays.^39^

While sugar-feeding and mating behaviours are attractive targets for developing new vector control strategies (box 3), these behaviours do not directly mediate malaria transmission so they have received little research attention and remain poorly understood.^40^ The full potential for epidemiological impact and optimal delivery practices for these strategies remains unclear. Strategic investment is needed to develop these intervention strategies, as well as the supporting knowledge base regarding the fundamental biology of malaria vector mosquitoes.^40^

Box 3 **Emerging opportunities for targeting adult mosquitoes when they seek sugar or mates**

- *Attractive toxic sugar baits (ATSBs)*: All mosquito species exploit sugar as a source of adult nutrition, so existing ATSB products designed for other insects may be highly effective for population control of malaria vectors. Additionally, home-made prototypes, constructed in situ with materials that are readily available in most African villages, have performed remarkably well against malaria vector populations in the few small-scale trails completed until now.^5^ ^78^ While some concerns with respect to impacts on non-target organisms persist, formulations using only environmentally friendly natural products are now becoming available.^79^ ^80^
- *Targeted space spraying of mating swarms*: Male mosquitoes aggregate in swarms, where they compete for the attention of female mosquitoes visiting them in search of a mate, usually for an hour or less at dusk or dawn. Mating swarms have been documented for several important malaria vector species all across Africa.^81^ One of the considerable advantages of spraying mating swarms is that they occur during specific, predictable, limited time periods, at dawn and/or dusk, at repeatedly reused locations that can be mapped.^81^ Preliminary, village-scale trials of manually targeting these aggregations with hand-held insecticide aerosol spray cans in Burkina Faso have yielded impressive early results,^39^ suggesting an exciting potential LMIC malaria control application for the well-established ground-based space-spraying technologies used so widely in HIC mosquito control programmes.

Box 4 **Examples of successful large-scale vector control programmes developed through ‘learning-by-doing’ strategies, following direct transition to phase IV effectiveness evaluations without any intermediate phase III efficacy studies.**

Against malaria vectors specifically:

- Numerous larval source management (LSM) programmes in the first half of the 20th century, including several successes in tropical of Africa, Asia and Latin America.^9^ ^10^
- Indoor residual spraying (IRS) against malaria vectors as the primary vector control measure of the Global Malaria Eradication Program in the second half of the 20th century, which enabled elimination of malaria from dozens of previously endemic countries.^82^
- Largely spontaneous and horizontal uptake of mosquito-proofed housing to protect many populations of the world during the first half of the 20th century, including expatriates living in what we now call low-income and middle-income countries (LMICs), canal workers in Panama and residents of southern USA.^32^ ^33^
- The systematic, vertical, proactive and decentralised local mosquito population abatement programmes established in high-income countries, especially the USA and Australia,^7^ ^8^ and to a far lesser extent in LMICs,^9–11^ over the past half century.

Against vectors of other pathogens:

- The Onchocerciasis Control Program spanning 10 west African countries in the latter quarter of the 20th century, which relied primarily on LSM of blackflies in rivers.^83^
- Southern Cone Initiative to eliminate American trypanosomiasis through IRS against triatomine bugs living inside houses.^84^
- Several large-scale, vertical Tsetse control programmes scattered across Africa, deploying varying combinations of attractive traps and targets with area-wide, outdoor space and surface spraying.^85^

### Programmatic, evidence-based development of new vector control strategies

A range of existing products and promising prototypes are now available that could be rapidly adapted for malaria vector control in LMICs (boxes 2 and 3). After decades of reliance on prescriptive ‘one size fits all’ global policies and pessimism about what is feasible in LMICs, the time has now come to begin developing the full diversity of available intervention opportunities. So how will countries adopt new strategies and technologies in the absence of definitive epidemiological evidence of efficacy, including rigorously controlled phase III efficacy trials? Fortunately, the historical evidence base (box 4), and now the WHO policy,^19^ are both firmly supportive of a ‘learning-by-doing’ strategy, rather than waiting for the evidence base to catch up with programmatic needs. With the exception of the recent LLIN scale-up, the most successful vector control programmes in history, including IRS and LSM for malaria prevention, were established and developed through programmatic phase IV monitoring, evaluation and operational research, without any preceding phase III trials (box 4). Such programmatic evaluations should begin at modest pilot scales, with rigorous epidemiological and entomological evaluation, as well as embedded operational research, followed by a progressive, rational roll-out as the necessary technologies, systems and supporting evidence mature.^5^ ^19^

Importantly, none of these exciting new vector control interventions will provide a universally applicable panacea; one size will not fit all. Different combinations of approaches will be needed in different geographies based on local ecology and vector biology, malaria epidemiology and operational capacity. While the need for national malaria control programmes to record epidemiological indicators of impact is obvious, programmes should also measure simple but robust indicators of mosquito and human behaviours that create these intervention opportunities, so that the best options can be selected, optimised and combined.^5^ ^41^ ^42^

### Emerging opportunities for financing more ambitious malaria vector control strategies in lower income settings

Ambitions to provide vertical mosquito population control services already have political support in some LMICs, to the extent that they finance them from predominantly domestic sources.^9–11^ Substantial international financing mechanisms already exist for improved housing, pharmaceutical supply and veterinary extension services in LMICs, all of which could be leveraged to support malaria vector control. Furthermore, a well-developed array of products with established high-value markets exist for personal protection, veterinary health and construction materials in HICs. Such commodities could be exploited to leverage subsidisation or donation for malaria control in LMICs, in the same way that curative drugs for several neglected tropical diseases of humans are procured.^43^ For example, donations of short-acting oral formulations of ivermectin are already used for eliminating onchocerciasis endoparasites from human populations, but longer lasting veterinary formulations for killing ectoparasites of livestock could also be used to suppress zoophagic populations of malaria vectors through agricultural extension systems that already exist in many LMICs.^5^ ^44^ Similarly, construction of mosquito-proofed houses and vertically delivered mosquito control are both well-established industries in HICs, for which creative financing mechanisms to support application in LMICs could be created.

## Conclusions

Malaria control is at an important historical juncture, as global progress over recent years is documented^1^ and attention turns to the 2030 elimination and 2040 eradication goals.^3^ ^4^ Recognising the challenges facing malaria vector control, including residual transmission, insecticide resistance and operational constraints to LLINs and IRS effectiveness, the WHO has acknowledged the need to overhaul the portfolio of vector control options available to national malaria control programmes.^19^ Nevertheless, the term *residual transmission* was only formally defined in 2016,^26^ and the Global Technical Strategy for Malaria up to 2030^4^ only mentions it in passing, without transparently acknowledging it as a fundamental, purely biological limitation to the level of impact that can be reasonably expected of LLINs or IRS.^5^ ^6^ Global policy now needs to consistently and unambiguously realign with both the programmatic needs and biological realities of malaria vector control, to unambiguously prioritise accelerated development of the diverse options available for malaria vector control in LMICs.

At present, the evidence base relating to these additional vector control options is lacking both in quantity and quality with respect to their application to prevention of malaria and other mosquito-borne pathogens.^45^ ^46^ Developing such an expanded toolbox for malaria vector control will require investment in product and system development, high-quality evaluations of efficacy and effectiveness, and operational research to define best practices for programmatic use of these additional interventions.
